# CdSe/ZnS quantum dots as a booster in the active layer of distributed ternary organic photovoltaics

**DOI:** 10.3762/bjnano.15.14

**Published:** 2024-02-02

**Authors:** Gabriela Lewińska, Piotr Jeleń, Zofia Kucia, Maciej Sitarz, Łukasz Walczak, Bartłomiej Szafraniak, Jerzy Sanetra, Konstanty W Marszalek

**Affiliations:** 1 AGH University of Krakow, Institute of Electronics, 30 Mickiewicza Ave, 30-059 Krakow, Poland; 2 AGH University of Krakow, Faculty of Materials Science and Ceramics, Department of Silicate Chemistry and Macromolecular Compounds, 30 Mickiewicza Ave, 30-059 Krakow, Poland; 3 R&D Department, PREVAC sp. z o.o., Raciborska 61, 44-362 Rogów, Poland; 4 retired, formerly: Cracow University of Technology, Institute of Physics, ul. Podchorążych 1, 30-084 Kraków, Poland

**Keywords:** efficiency, luminescence, organic solar cells, quantum dots

## Abstract

Organic solar cells are a promising candidate for practical use because of their low material cost and simple production procedures. The challenge is selecting materials with the right properties and how they interrelate in the context of manufacturing the device. This paper presents studies on CdSe/ZnS nanodots as dopants in a polymer–fullerene matrix for application in organic solar cells. An assembly of poly(3-hexylthiophene-2,5-diyl) and 6,6-phenyl-C71-butyric acid methyl ester was used as the active reference layer. Absorption and luminescence spectra as well as the dispersion relations of refractive indices and extinction coefficient were investigated. The morphologies of the thin films were studied with atomic force microscopy. The chemical boundaries of the ternary layers were determined by Raman spectroscopy. Based on UPS studies, the energy diagram of the potential devices was determined. The resistivity of the layers was determined using impedance spectroscopy. Simulations (General-Purpose Photovoltaic Device Model) showed a performance improvement in the cells with quantum dots of 0.36–1.45% compared to those without quantum dots.

## Introduction

Organic solar cells have been intensively developed in recent years as the third generation [1–2] of photovoltaic cells, next to dye-synthesized solar cells and perovskite cells. One of the relatively novel concepts of organic solar cells that yield higher efficiencies is ternary organic cells [3–6]. The idea of ternary organic cells is to introduce additional material (donor or acceptor) into a typical donor–acceptor active layer in the bulk heterojunction (BHJ). This arrangement is supposed to combine the absorption of the three components to obtain outstanding excitation and optimum charge transport in the mixture. Small-molecule materials, dyes, polymers, fullerenes, and ligands have been introduced as a third component so far. Quantum dots (QDs) are also beneficial materials for ternary solar cells.

QDs and nanoparticles as zero-dimensional materials are used in opto- and nanoelectronics. QDs establish a class of materials transitional between subatomic and mass types of matter. The classification of QDs according to the core material is divided into cadmium [7–8], silver [9–10], indium [9], carbon [11–12], and silicon [13–14]. Numerous nanodot applications include quantum performance enrichment for diodes [15–16], memory devices [17–18], transistors [19–20], and solar cells. They are also widely used in biological applications [21–23]. QDs have been used in various functions of solar cells, including electron- or hole-transporting layers [24], active absorbing layers, and other components [25–26]. Inorganic quantum dots are considered substitutes for fullerene acceptors. Their biggest advantages are a tunable band gap, various absorption spectra, and comparatively high mobility of carriers [27–28]. The application of quantum dots and nanoparticles in organic solar cells has already been demonstrated [29–34]. The list of both materials and types, as well as applications, is not limited. Moreover, new quantum dots with innovative properties are still being researched and produced [35–37].

This publication aims to validate the potential of CdSe/ZnS quantum dots for the enrichment of the active layer and, ultimately, for application in the active layer of organic BHJ cells. An illustrative QD synthesis was described by Dabbousi and co-workers [38]. Methods and approaches were intensively developed [39–40]. One of the main existing challenges in synthesizing QDs is to increase their photoluminescence efficiency while simultaneously shifting the photoluminescence maximum to longer wavelengths. Initial applications focused on OLEDs. CdSe/ZnS quantum dots are luminescent inorganic nanostructures. The emission wavelength is determined by the crystal size and structure; crystals of the same chemistry can have emission maxima spanning a wide range. As the size of the dots decreases, both their optical absorption and emission shift to higher energies [41]. Because hydrophilic QDs are coated with –COOH groups, they can be easily labeled for chemical and biological applications. Low-molecular-weight organic molecules coat highly luminescent semiconductor nanocrystals.

## Materials and Methods

**Figure 1:**
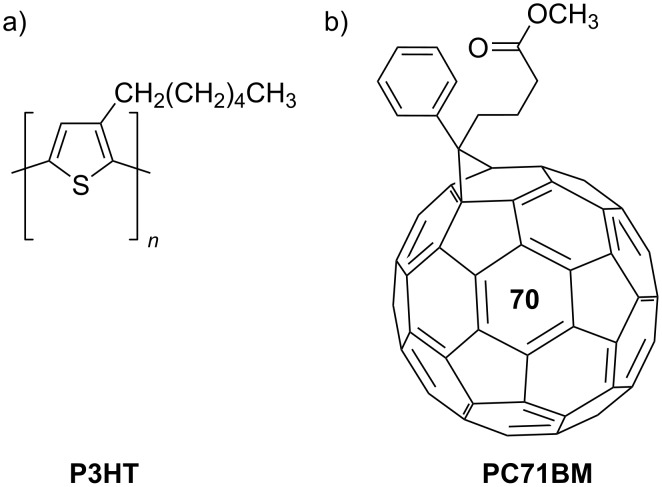
Chemical formulas of the base of the active layer. (a) Donor: poly(3-hexylthiophene-2,5-diyl) (P3HT) and (b) acceptor: 6,6-phenyl-C71-butyric acid methyl ester (PC71BM).

Thin films, the intended active layers, were fabricated using the spin-coating method. The layers were a mixture of donor and acceptor materials enriched with quantum dots. Poly(3-hexylthiophene-2,5-diyl) (P3HT, donor) and 6,6-phenyl-C71-butyric acid methyl ester (PC71BM, acceptor) are used as base materials and reference materials in organic photovoltaic cell research [42–44] (Figure 1). These compounds were supplied by Merck KGaA, Germany. The CdSe/ZnS quantum dots used in this investigation were commercially purchased (PlasmaChem GmbH, Germany).

Materials were provided in the solid state; the solvent used in the experiments was of spectroscopic grade. The resulting quantum dot solutions were homogeneous, transparent, and colored, except for QD640, which was a dark solution with only little transparency. First, P3HT (regioregular) and PC71BM were dissolved in chloroform to obtain 10 mg/mL solutions. The solutions were combined with the quantum dots solution in selected ratios. The mixtures were ultrasonicated for 20 s and then applied to the substrate according to the test method. The angular velocity of the spin coater was also determined by the specific test method and its required film thickness. To keep the typical ratio of the donor and acceptor equal to 1:1 and simultaneously highlight the QD impact on the layer under consideration, a weight ratio of 1:0.5:0.5 was chosen (donor:acceptor:QDs). The quantum dot designations used in the paper are summarized in Table 1. The numbers next to the abbreviation QD refer to the maximum luminescence suggested by the manufacturer. The size of the nanoparticles, including the size of the shell (0.6 nm), and the core size declared by the manufacturer are included in Table 1.

**Table 1:** Designations of the nanodots used in the system.

Designations	Nanoparticles	Declared emission [nm]	Core thickness [nm]	QD thickness [nm]

QD480	ZnCdSe/ZnS	480	2.3	2.9
QD520	CdSe/ZnS	520	2.7	3.3
QD580	CdSe/ZnS	580	3.6	4.2
QD600	CdSe/ZnS	600	4.2	4.8
QD640	CdSe/ZnS	640	6.2	6.8

UV–vis spectroscopy was conducted with an Avantes Sensline Ava-Spec ULS-RS-TEC fiber-optic spectrophotometer and an Avantes AvaLight DH-S-BAL-Hal lamp. The absorption and luminescence spectra of a solid were studied as a thin film deposited on quartz, and measurements in solution were obtained in chloroform. The wavelength ranged from 250 to 1000 nm.

A Woolam M-2000 ellipsometer was used for spectroscopic ellipsometry analysis. The measurement range was 350–1650 nm. The measurement was carried out for three incidence angles (65°, 70°, and 75°).

A Bruker atomic force microscope (AFM) MULTIMODE 8 was used in the measurements in the ScanAsyst in Air mode, using silicon nitride probes (with a nominal tip radius of 2 nm and a spring constant equal to 0.4 N/m). The substrate was monocrystalline silicon. A WITec Alpha 300 M+ spectrometer with a 488 nm laser, 600 groove grating, and a 100× ZEISS objective was used for Raman measurements. The samples were deposited on a glass substrate. Ultraviolet photoelectron spectroscopy (UPS) was conducted in an ultrahigh-vacuum chamber with a base pressure of approximately 8 × 10^−10^ mbar, using the XPS/UPS/ARPES PREVAC setup. The analysis chamber was equipped with a PREVAC Ea15 hemispherical analyzer and a PREVAC EA15 40B source (UV power *U* = 0.56 kV, *P*_UV_ = 55 W, He I). The beam energy scale was calibrated at the Fermi level of 16.87 eV. The samples were applied to gold foils. A system equipped with a Solartron 1260A frequency response analyzer, a Solartron 1296A dielectric interface, and a supervisory computer was prepared to determine the impedance parameters of the obtained structures. The impedance characteristics of the obtained samples were determined in a wide frequency range from 10^−1^ to 10^6^ Hz at room temperature. The amplitude of the alternating current signal was set to 0.1 V. The ZView software was used to analyze the recorded impedance spectra.

## Results and Discussion

### UV–vis spectroscopy

**Figure 2:**
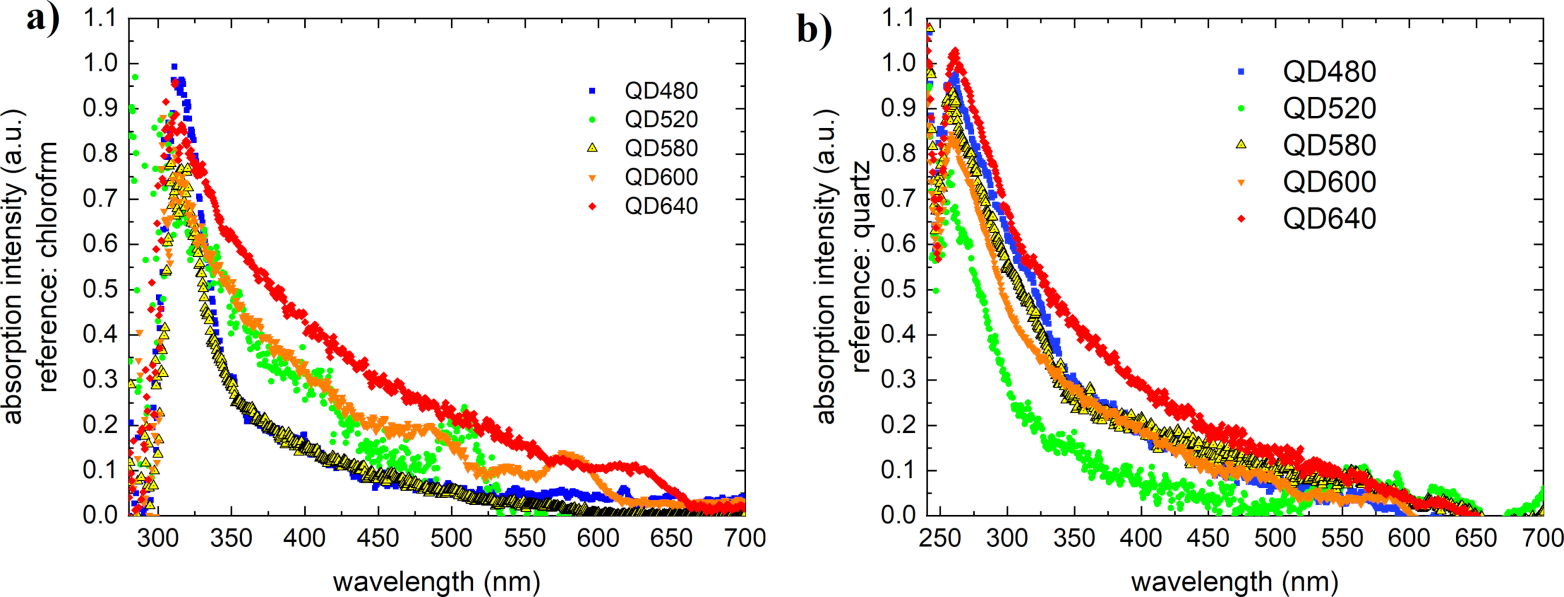
QD absorption spectra from (a) solid-state measurements and (b) solution measurements (b).

The pure quantum dots exhibit absorption in the ultraviolet with a small exponentially decreasing band up to 500–600 nm (Figure 2). The absorption band maximum is about 260 nm in the solid state (quartz was used as reference) and 330 nm in chloroform solution.

**Figure 3:**
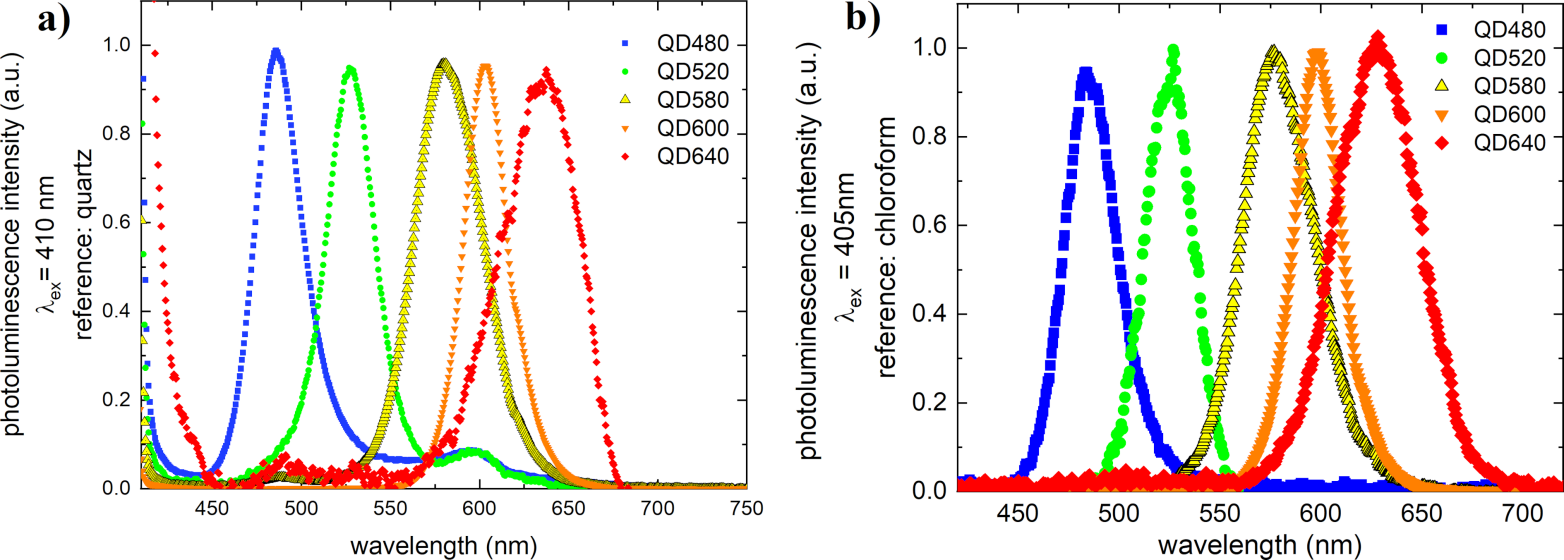
QD photoluminescence spectra investigated in (a) solid-state measurements and (b) solution measurements.

The photoluminescence spectra, excited by a 405 nm laser, are shown in Figure 3. This type of absorption almost coincides with the absorption spectrum of PC71BM fullerenes. It follows from this that not much can be changed in the system when it concerns the additivity of absorption.

The luminescence maxima were as follows for the solid state and the solutions: 484 nm for QD480, 526 nm for QD520, 583 nm for QD580, 596 nm for QD600, and 636 nm for QD640. The full width at half maximum values of the fluorescence peaks increase, and they are about 30–40 nm. The luminescence peak for QD640 is significantly wider at about 50 nm.

**Figure 4:**
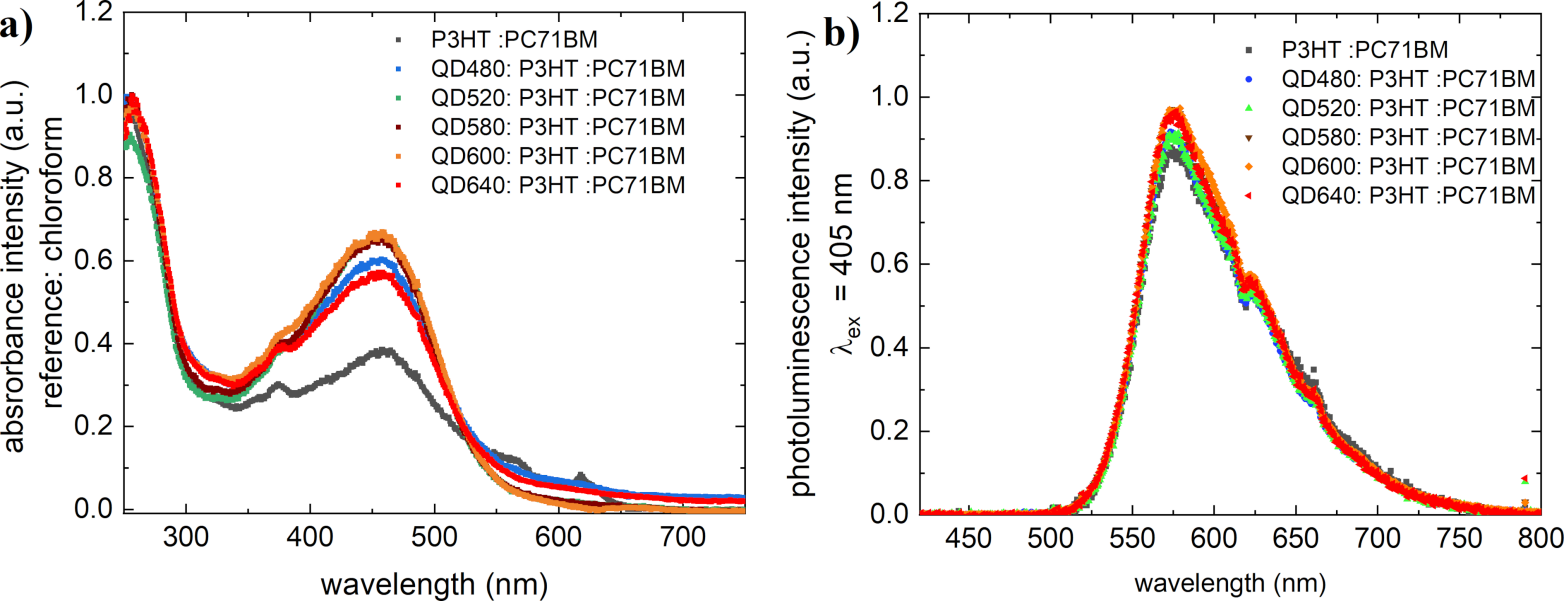
(a) Absorption spectra and (b) photoluminescence spectra of mixtures of quantum dots:P3HT:PC71BM mixtures.

Figure 4 shows the absorption and luminescence spectra of mixtures of P3HT:PC71BM:QDs (ratio 1:0.5:0.5) measured in solution. In the case of absorption, spectrum shape and absorption range remain unchanged. The addition of quantum dots slightly increases the intensity in the range from 350 to 550 nm compared to the base P3HT:PC71BM array. In the case of photoluminescence spectra, the effect of quantum dots in the spectrum is not apparent, indicating that the transitions in the mixture are quenched. This suppression is a result of energy transfer, which necessitates electron clouds of atoms or chemical groups to exhibit overlapping regions. During energy transfer, an electron moves from the donor to the acceptor and occupies the acceptor’s least occupied orbital. As a consequence of this energy transfer, the donor returns from an excited state to its ground state, while the acceptor remains in its ground state due to its higher electronegativity. This absence of excited fluorophores emitting light signifies the occurrence of the quenching process.

### Spectroscopic ellipsometry

Spectroscopic ellipsometry studies were performed to determine changes in optical performance with temperature. A multilayer model with a silicon substrate layer was fitted to the obtained Ψ and Δ values (point by point). The layer under consideration was first pure quantum dots and then a ternary system with PC71BM and P3HT. The refractive index (*n*) of the quantum dots thin film and the extinction coefficient (*k*) were estimated using the Lorentz model modified by Tauc. The complex dielectric function is related to the complex refractive index *n* by the equation in the Tauc–Lorentz model:


[1]

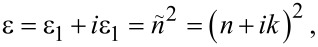



where ε_1_, ε_2_ are, respectively, the real and imaginary parts of the dielectric function, *n* is the refractive index, and *k* is the extinction coefficient.

The real part of the Tauc–Lorentz dielectric function is given as:


[2] $$\varepsilon_{1}\left(E\right)=n^{2}\left(E\right)-k^{2}\left(E\right).$$

The imaginary part function is described by:


[3] $$\varepsilon_{2}\propto \frac{{\left(E-E_{g}\right)}^{2}}{E^{2}},$$

[4] $$\varepsilon_{2}\left(E\right)=\frac{AE_{0}\Gamma {\left(E-E_{g}\right)}^{2}}{E\left[{\left(E^{2}-E_{0}^{2}\right)}^{2}-\Gamma^{2}E^{2}\right]}\Theta \left(E-E_{g}\right),$$

where *E*_g_ is the band gap of the material, *E*_0_ is the peak in the joint density of states, Θ is the Heaviside theta function, Γ is the broadening parameter, and *A* is a prefactor.

**Figure 5:**
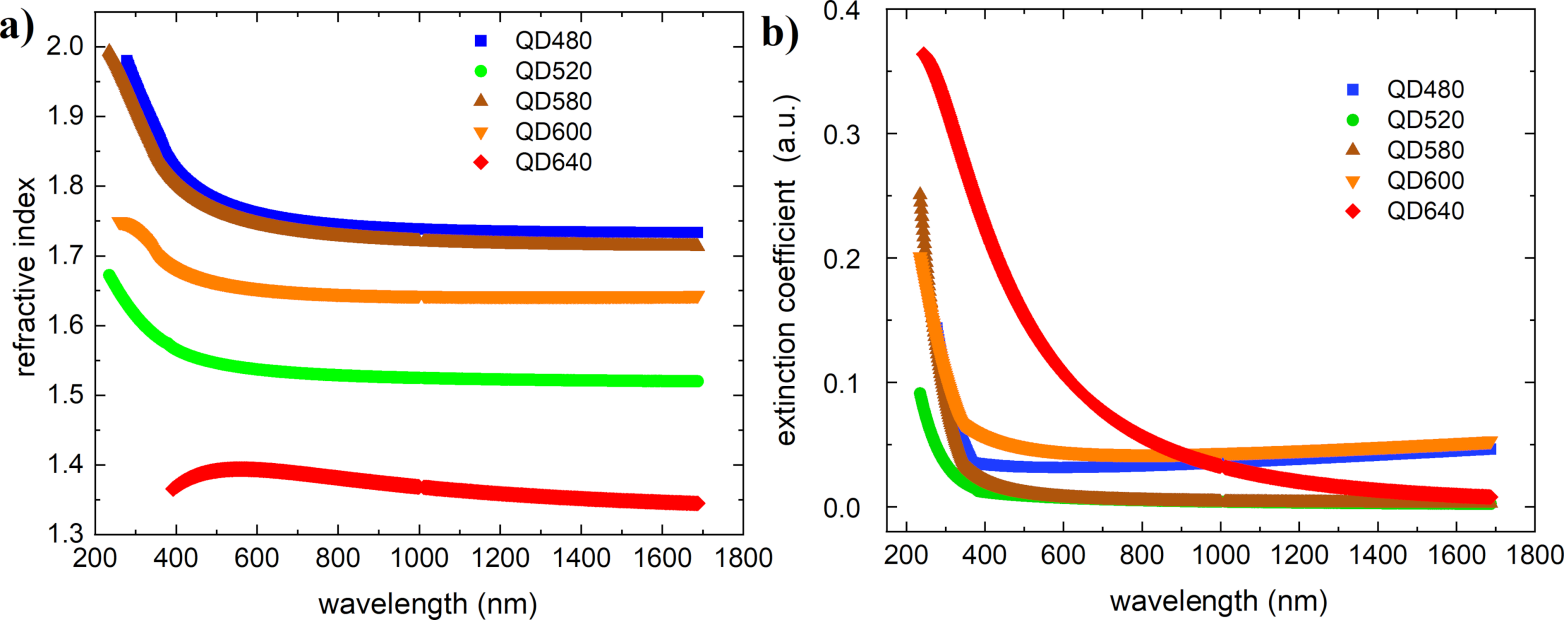
(a) Refractive indices and (b) extinction coefficients of the investigated quantum dots.

**Figure 6:**
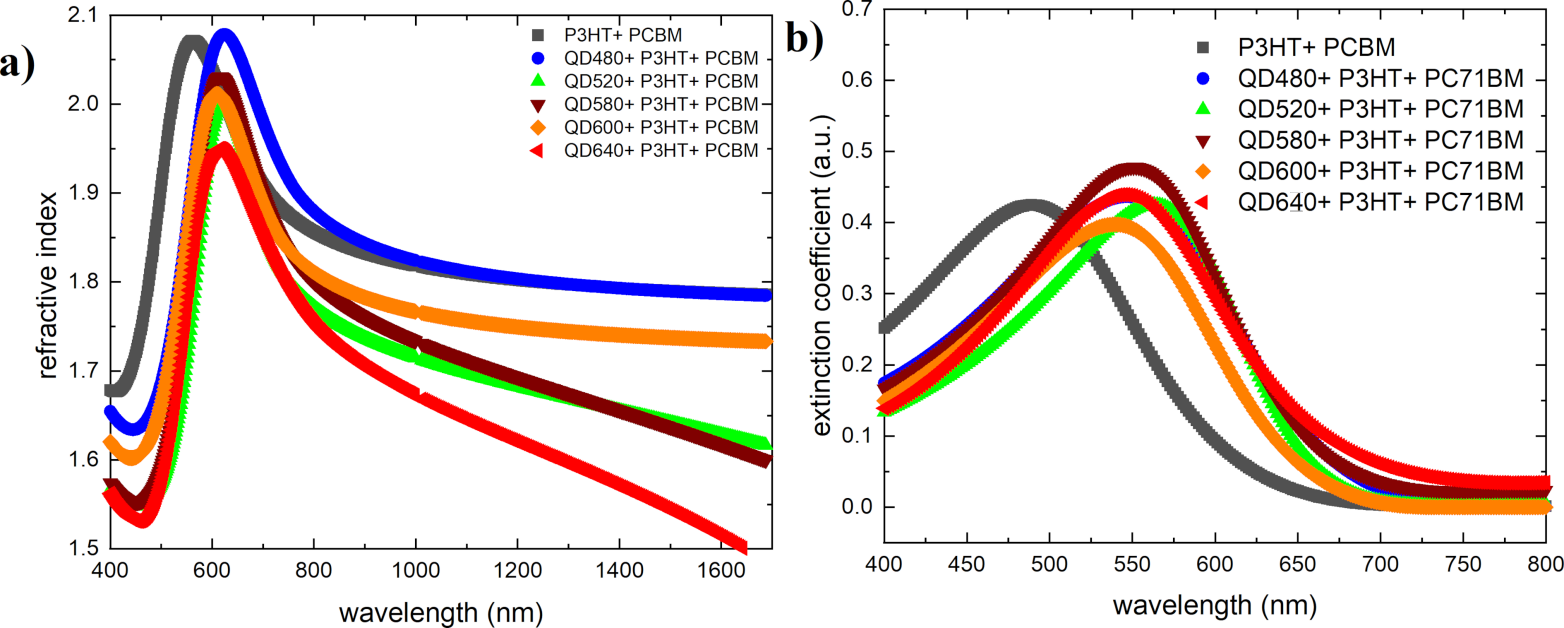
(a) Refractive indices and (b) extinction coefficients of quantum dots+P3HT+PC71BM mixtures.

The refractive indices and extinction coefficients as functions of the wavelength for the materials tested and the ternary mixtures are shown in Figure 5 and Figure 6, respectively. The acquired values of *n*(λ) and *k*(λ) satisfy the Kramers–Kronig relations.

As evidenced by the spectrum, QD640 has a different waveform than the other quantum dots. It is smoother for the extinction coefficient and shifts the maximum toward short wavelengths for the refractive index. Refractive indices and extinction coefficient parameters for investigated quantum dots thin films are summarized in Table 2.

**Table 2:** Refractive indices and extinction coefficient parameters for investigated quantum dots thin films.

Quantum dots	Refractive index range	Refractive index at 633 nm	Extinction coefficient maximum (arb. un.)	Extinction coefficient at 633 nm (arb. un.)

QD480	2.00–1.75	1.76	0.151	0.0333
QD520	1.68–1.51	1.54	0.0922	0.00631
QD580	2.00–1.74	1.75	0.253	0.0122
QD600	1.75–1.66	1.65	0.200	0.0441
QD640	1.40–1.35	1.39	0.364	0.100

In ternary systems, a shift of the extinction coefficient maximum toward lower energies can be observed, thus approaching the maximum of the sunlight spectrum (approximately 550 nm). The refractive index of the thin film for the ternary mixture enriched by QD480 increases slightly compared to the base mixture P3HT+PC71BM. For the other mixtures, there is a decrease in the magnitude of the maximum refractive index, which decreases with the increasing fluorescence wavelength of the quantum dots studied. The paper by Dement et al. [45] also presents elipsometric studies, however in a narrower scope. Obtaining quantum dots was done by a different method. Nonetheless, they obtained absorption features not observed in our study.

### Atomic force microscopy

**Figure 7:**
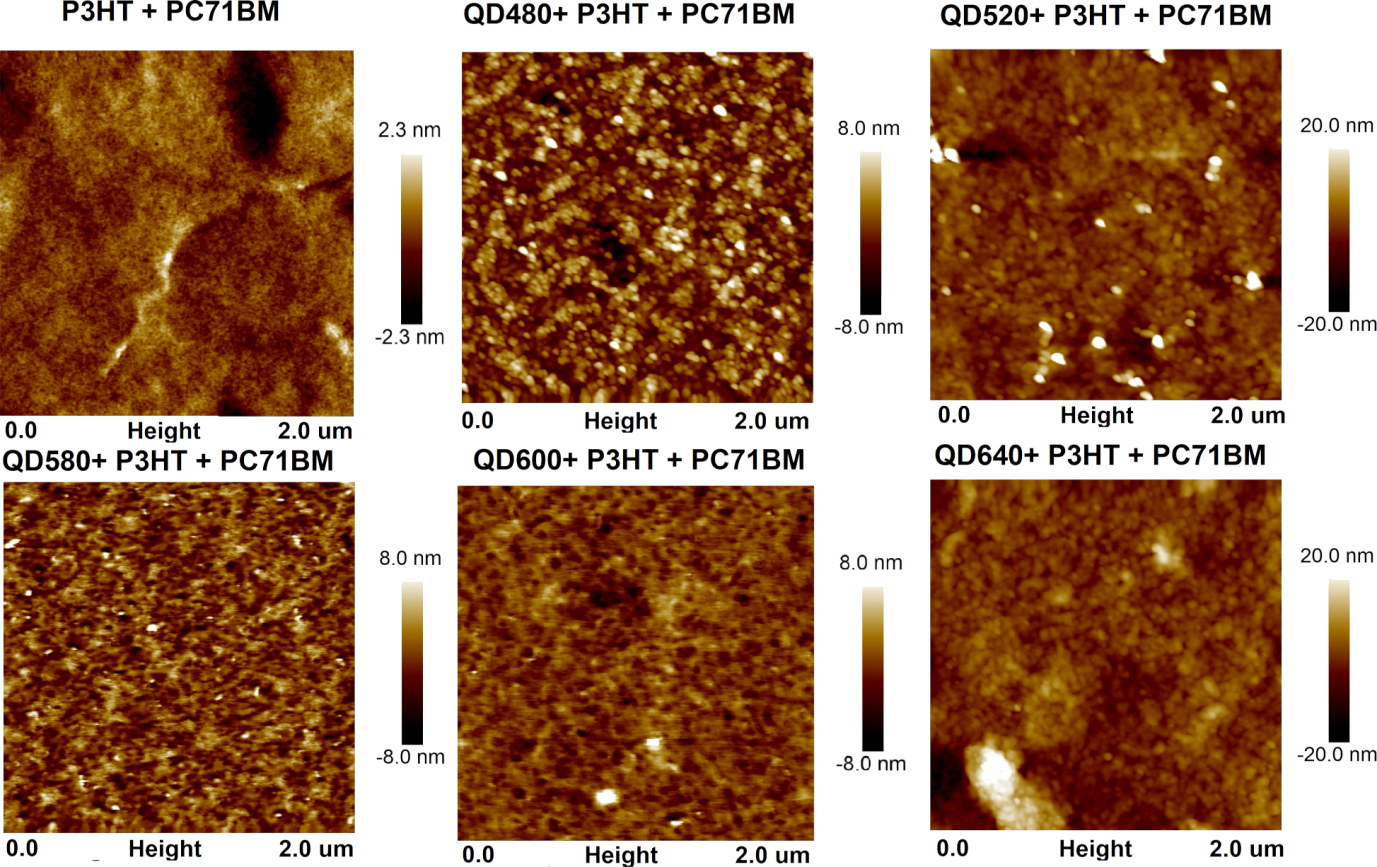
AFM 2D images of quantum dots+P3HT+PCBM mixtures.

Surface examinations of the sample mixtures were performed. Figure 7 illustrates the surface morphology in a two-dimensional format. Three-dimensional images of the surface are in Supporting Information File 1, Figure S1.

The roughness profile parameters for the obtained layers are summarized in Table 3. *R*_a_ is the arithmetic average of the profile height deviations from the mean line, *R*_q_ is the root mean square average of the profile height deviations from the mean line, and *R*_max_ is the distance between the lowest and highest points of the investigated profile.

**Table 3:** Roughness indices *R*_q_, *R*_a_, and *R*_max_ of the investigated layers.

Sample	*R*_q_ [nm]	*R*_a_ [nm]	*R*_max_ [nm]

P3HT+PC71BM	0.523	0.381	5.66
QD480+P3HT+PC71BM	2.30	1.83	23.2
QD520+P3HT+PC71BM	4.20	2.64	60.6
QD580+P3HT+PC71BM	2.04	1.59	24.9
QD600+P3HT+PC71BM	1.58	1.15	36.9
QD640+P3HT+PC71BM	4.21	2.92	42.9

The addition of quantum dots to the layer increases its roughness parameters. The surface also shows greater morphological differentiation in the form of numerous peaks present throughout the surface. The third component in the BHJ for the systems studied so far supported a reduction in roughness, which favored the charging process of the metallic electrode. These were used to facilitate the transport of charge carriers between the electrode and the layer, which can become problematic because of limitations in the conductivity of organic materials. The changes in roughness presented from the AFM results due to dot doping align with the results presented in the work of Cha et al. [46], Lewińska et al. [47], and Ohring [48]. By design, quantum dots are supposed to play a different role as transport enhancers with their properties.

### Raman spectroscopy

**Figure 8:**
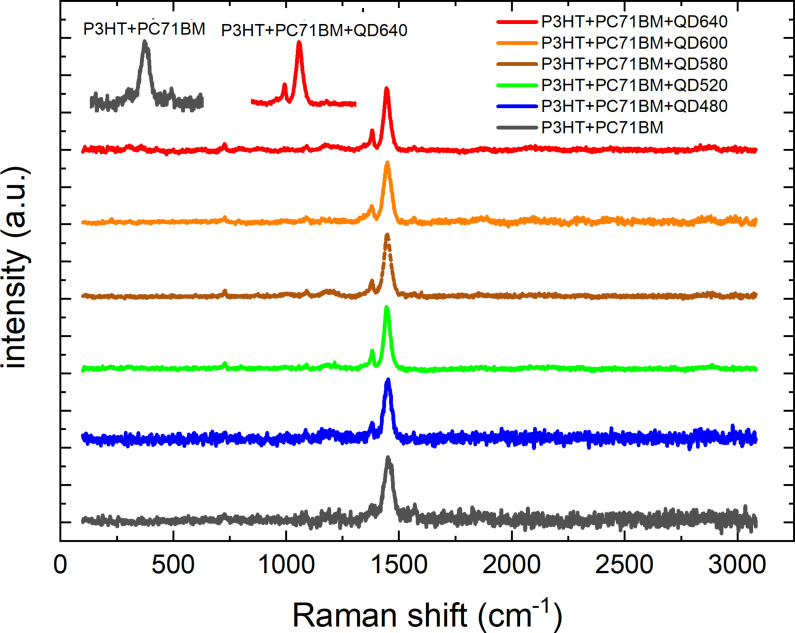
Raman plots of P3HT, PCBM, and QDs+P3HT+PCBM mixtures.

Raman measurements for P3HT and PCBM were recorded previously [49]. The C–S–C ring deformation (725 cm^−1^), C–C intra-ring stretching (1,379 cm^−1^), and symmetric C=C stretching vibrations (1,447 cm^−1^) of P3HT were outlined. The PCBM Raman spectrum has four peaks at 658, 1,038, 1,127, and 1,570 cm^−1^. Raman spectra for the mixtures considered are shown in Figure 8. Because the intensity of the P3HT peaks is much higher than that of the PCBM peaks, only the P3HT peaks are visible in the mixture [50]. It can be seen that for the reference, the layer shows an additional peak at 1,468 cm^−1^. For the doped systems, a peak at 1,378 cm^−1^ is also visible.

The donor–acceptor mixture is dominant in the system. Therefore, the peaks of the QD dopant are not visible. At the same time, it should be noted that in mixtures with quantum dots (except QD480), the clarity of the peaks increases, which may suggest an increase in the degree of crystallinity. Increased molecular order and increasing crystallinity of the system were positively correlated with changes in device performance [51].

### Ultraviolet photoelectron spectroscopy

**Figure 9:**
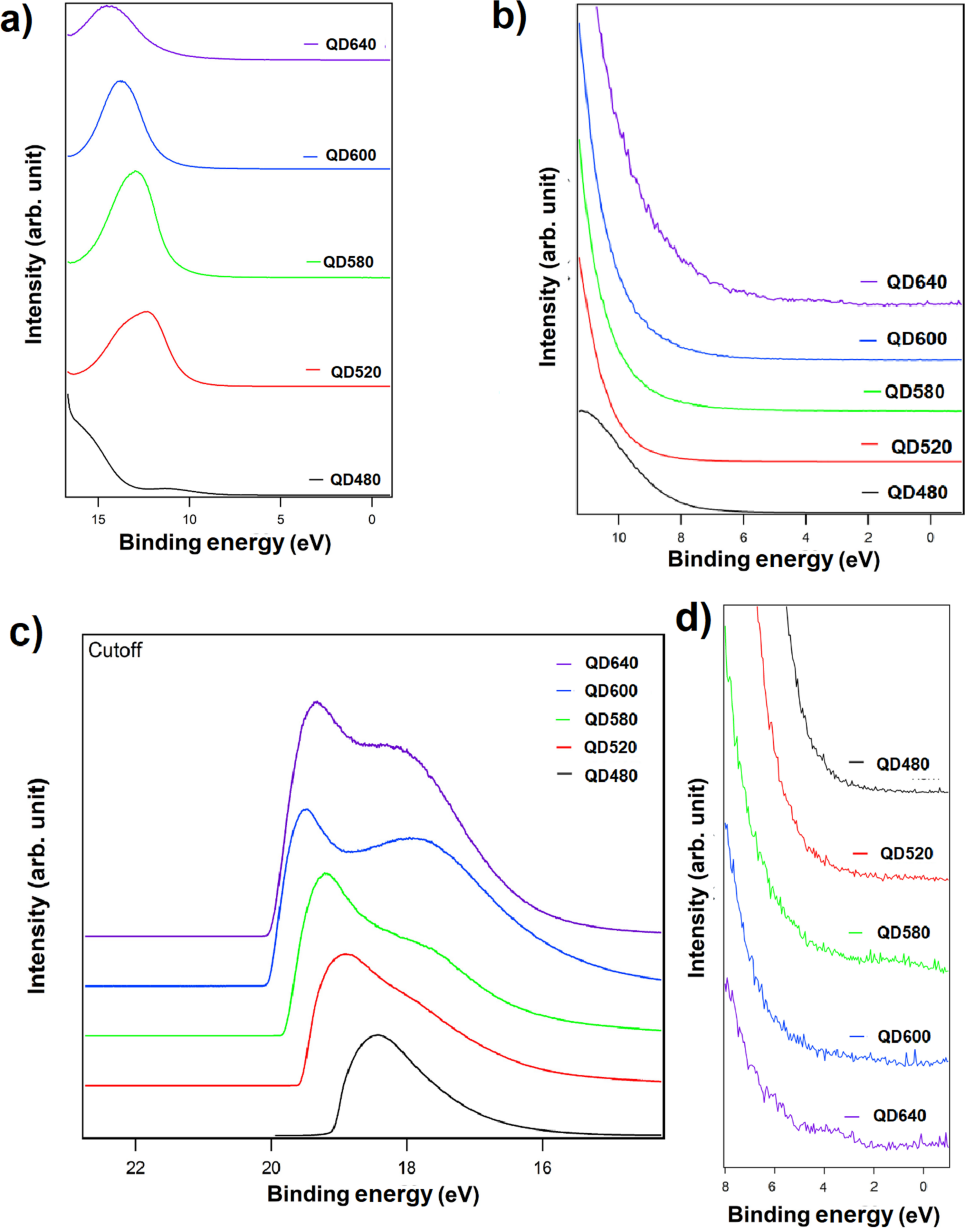
(a, b) UPS survey spectra, (c) comparison of the cutoffs, and (d) comparison of the UPS spectra of the valence bands.

We obtained survey spectra for the materials under consideration (Figure 9a,b). Figure 9c illustrates the obtained valence spectrum in various ranges, as well as a comparison of the cutoffs for all included samples. Spectra are shifted vertically for clarity.

No core–shell stratification of states was observed in the spectra for smaller quantum dots (QD480 and QD520). However, the influence of a changing core was observed for the other nanoparticles, where changes in the spectral maxima could be seen for energies around 16–17 eV. This indicates the different densities of states for the specific nanodots. Based on the cutoff of the lowest binding energy of the curve in the area (Figure 9d), the highest occupied molecular orbitals (HOMOs) have been determined. Levels have been calculated by subtracting from 21.2 eV (He I) the Fermi level position from the graph in Figure 9c and adding the Fermi level position relative to the HOMO from Figure 9d. The HOMO values obtained are −6.0 eV, −5.3 eV, −6.0 eV, −5.7 eV, and −4.4 eV for QD480, QD520, QD580, QD600, and QD640, respectively.

According to Hummon et al. [52] the CdSe valence-band edge was determined to be −6.8 eV, from thin-film UPS and photoluminescence measurements. The CdSe conduction-band edge was determined by the photonic band gap (2.0–4.8 eV). The valence- and conduction-band edges of the ZnS shell, −7.4 eV and −3.4 eV, respectively, were also determined. These results in the context obtained by us are reasonable. There was also a DFT study conducted [53], however, for crystalline CdSe/ZnS variants , where the energy gap obtained was equal to 2.69 eV.

### Impedance spectroscopy

ZView software was used to analyze the frequency characteristics. Substitute circuit models for the obtained thin films were proposed. The values of individual model parameters are summarized in Table 4.

**Table 4:** The parameters values obtained for the impedance spectroscopy model for cells with nanoparticles (QD520, 580, 600:P3HT:PC71BM) and reference cell P3HT:PC71BM.

Sample	*R* [Ω]	CPE-T [F]	CPE-P [Phase]

QD520:P3HT:PC71BM	2203	1.50 × 10^−8^	0.93
QD580:P3HT:PC71BM	3732	9.94 × 10^−7^	0.81
QD600:P3HT:PC71BM	4809	3.75 × 10^−8^	0.95
P3HT:PC71BM	230 750	2.28 × 10^−7^	0.80

The replacement models were made up of *R*-elements and a constant-phase element (CPE), which are connected in parallel based on the fit results. The CPE is an element generating impedance with a constant phase angle in the complex plane. The P (phase) of the CPE element (CPE-P) for the analysis varies from 0.8 to 0.95, which is responsible for the capacity. The element T (capacitance) of the CPE (CPE-T) is responsible for the polarization of the sample in various areas of the material structure and on the electrodes. The mechanisms of ionic or electronic conductivity are represented by the resistance *R* [54].

Substitute systems with nanodots QD520:P3HT:PC71BM, QD580:P3HT:PC71BM, and QD600:P3HT:PC71BM are characterized by significantly lower resistance compared to P3HT:PC71BM, which may be responsible for the higher ionic or electronic conductivity of these layers. A similar effect was reported by Zhao et al. and Zang and co-workers [55–56].

### Simulations and analyses of ternary OPVs

**Figure 10:**
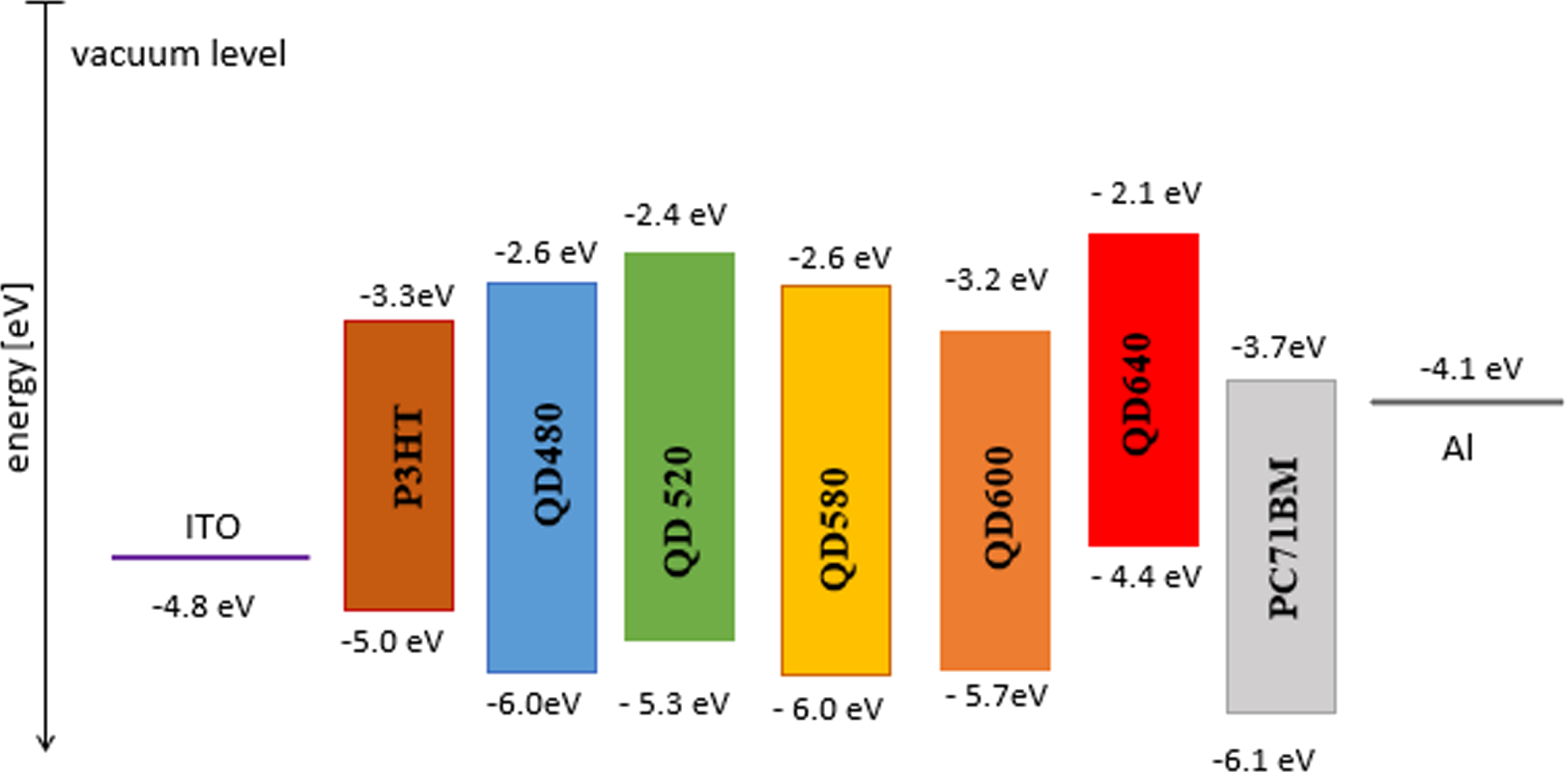
Energy diagram for potential ternary solar cells.

The energy diagram of a potential solar cell with aluminum and indium tin oxide (ITO) electrodes is presented in Figure 10. The HOMO was determined using the UPS spectrum of the valence bands for all samples.

The lowest unoccupied molecular orbital (LUMO) was determined by subtracting the energy gap from the HOMO level. The energy gap was obtained from the Tauc diagram [57]. The levels correspond to a mixture of the levels obtained for pure CdSe and ZnS [58].

To evaluate the potential applicability of ternary mixtures, the volume heterojunctions of ITO/PEDOT:PSS/active layer/aluminum and ITO/active layer/aluminum systems were simulated using the computational tool “General-Purpose Photovoltaic Device Model (GPVDM)” [59]. This software was proofed functional for similar organic systems [60]. The active layer was a mixture of a polymeric donor and P3HT, with a PCBM fullerene acceptor doped with appropriate QDs in ternary systems. Information about the materials taken from measurements was entered into the material database of GPVDM. A customized blend of P3HT:PC71BM was also used in the simulations.

**Figure 11:**
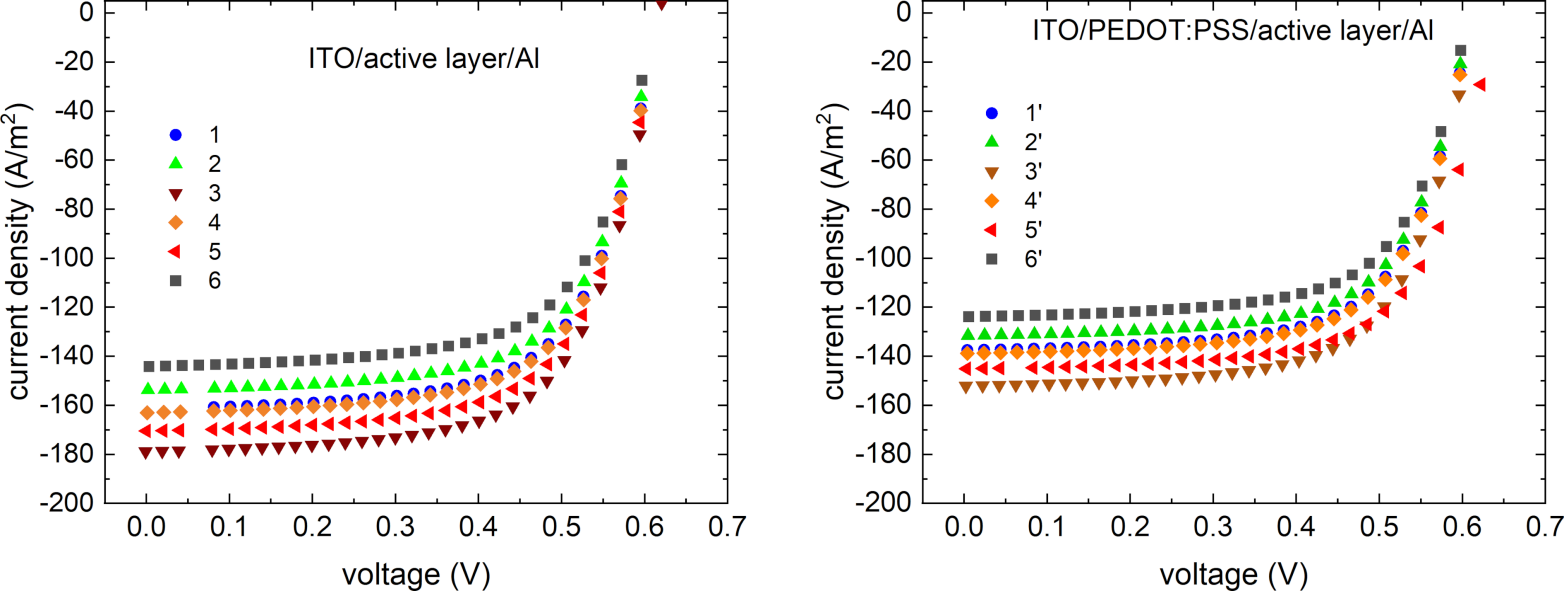
Current–voltage characteristics for (a) ITO/active layer/Al and (b) ITO/PEDOT:PSS/P3HT:PCBM:QD/aluminium bulk heterojunctions cells.

Simulations of current and voltage characteristics were performed (Figure 11). The change in performance of QD-doped devices was compared to the reference system (ITO/PEDOT:PSS/P3HT:PCBM/aluminium).

PEDOT:PSS ([poly(3.4-ethylene dioxythiophene] polystyrene sulfonate) is an intrinsically conductive polymer, a mixture of two ionomers, with a HOMO level equal to 5.17 eV [61]. It is widely used as a charge-transport and smoothing layer. Concerning the postulated use of nanorods as acceptors, weight ratios in the active layer of P3HT:PCB71M:QD of 2:1:1 were used so that the weight ratio of donor:acceptors is 1:1.

The device efficiency achieved in the simulations in both architectures was increased relative to the reference cells (6 and 6′ devices in Table 5). The fill factor for all devices with QDs remained essentially unchanged. The original reference cell efficiency obtained for materials from the program database was 4.63% (with PEDOT:PSS) and −5.24% without PEDOT:PSS. The open-circuit voltage varies in the millivolt region. The biggest change in performance concerned the short-circuit current.

**Table 5:** Simulated parameters obtained for photovoltaic cells.

Device number	Active layer	*J*_sc_ (A/m^2^)	*U*_oc_ (V)	FF (arb. un.)	Efficiency (%)

ITO/active layer/Al					

1	QD480:P3HT:PC71BM	−161.5	0.615	0.656	6.54
2	QD520:P3HT:PC71BM	−153.7	0.613	0.661	6.23
3	QD580:P3HT:PC71BM	−178.9	0.618	0.655	7.24
4	QD600:P3HT:PC71BM	−163.0	0.615	0.659	6.60
5	QD640:P3HT:PC71BM	−170.5	0.617	0.658	6.92
6	P3HT:PC71BM	−144.2	0.611	0.658	5.79

ITO/PEDOT:PSS/active layer/Al					

1’	QD480:P3HT:PC71BM	−137.5	0.666	0.666	5.58
2’	QD520:P3HT:PC71BM	−131.6	0.608	0.669	5.35
3’	QD580:P3HT:PC71BM	−152.1	0.612	0.663	6.19
4’	QD600:P3HT:PC71BM	−138.8	0.609	0.666	5.64
5’	QD640:P3HT:PC71BM	−145.8	0.611	0.666	5.91
6’	P3HT:PC71BM	−123.9	0.606	0.665	4.99

## Conclusion

Absorption spectra of the studied QDs indicate the role of a second acceptor (non-fullerene acceptor [62]) in the heterojunction volume. We may also consider QD materials as donors. By establishing the QD’s role as an acceptor, the QD additive is considered the first donor (with a higher LUMO level), and P3HT would be the second. The HOMO offsets [63] are 0.7 eV for QD480, 0.9 eV for QD520, 0.7 eV for QD580, 0.1 eV for QD600, and 1.7 eV for QD640. Based on the energy level system, it can be concluded that the best energy match in the donor:acceptor system is for QD600 dots. QD600 dots are the only material that can be considered in the system as a second donor in the energy diagram. The largest HOMO offset may explain the highest efficiency for the QD640-doped cell. The energy offset should be equal to or greater than the binding energy of singlet and triplet excitons. The energy levels system of the considered devices with QDs determines the optimal photocurrent of dissociation for most singlet excitons, which require at least 0.07 eV energy [64]. The production of charge-transfer carrier combinations as part of the donor:acceptor blend is prompted by singlet excitons. This increases the electron transfer current, leading to higher efficiency. By incorporating quantum dots into active layers, the following effects were observed: boosted exciton formation, minimized interface charge recombination, and increased charge transport. This was also demonstrated by Lv and co-workers [65]. The LUMO level of QD600 is lower than that of the donor P3HT, while the other QDs have higher LUMO levels. This difference in LUMO levels poses a challenge for the transportation of electrons to the cathode. Conversely, the HOMO levels of four QDs, namely QD480, QD250, QD580, and QD600, are higher than the HOMO level of P3HT. As a result, the positively charged holes generated can be effectively transported to the anode. The increase in efficiency due to the non-enrichment of absorption and the energy level system is mainly caused by the many times reduced resistivity, demonstrated by the impendance spectroscopy results.

Absolute values of efficiency growth for the investigated doped QD cells are about 1–2%. However, counting the percentage increase, these small values represent an increase of 10–20% over the reference cell. The literature reveals a wide range of performance values for organic cells doped with quantum dots. The efficiency of organic solar cells showed a boost of 3.08% when it featured optimal PbS colloidal quantum dots (CQDs). The architecture of the device was ITO/poly(3-hexylthiophene) (P3HT)/polymer-[6,6]-phenyl-C61-butyric acid methyl ester (PCBM)/Ca/Al [66]. With poly{[2,7-(9-(2-ethylhexyl)-9-hexylfluorene])-*alt*-[5,5-(4,7-di-2-thienyl-2,1,3-benzothiadiazole)]} (PFDTBT) quantum dots doped into organic solar cells, a maximum PCE equal to 6.81% was obtained, corresponding to a 23.8% enhancement compared with the undoped device [67]. CQD/polymer hybrid solar cells reached 12% efficiency [68]. The devices were used in a CQD/small molecule acceptor–bridge–polymer hybrid composition. For black phosphorous quantum dots (BPQDs) devices, the PCE was enhanced from 11.8 to 13.1% for PTB7-Th/FOIC based OSCs [69]. Perovskite cells with PbS QDs reached more than 10% [70–71]. For other types of QDs, perovskite cell technologies are also intensively developed [72–73]. QD-assisted organic cells also achieve better and better (even 15%) performance [68–69]. Nevertheless, understanding the mechanisms and improving the cells still requires extensive research.

## Summary

This study aimed to find out how doping active layers with a third component, specifically quantum dots, affects the properties of the layer applied in photovoltaic cells. Spectroscopic studies have been performed to establish absorption spectra and confirm excited states proven by luminescence. The dispersive spectra of the refractive index and extinction coefficient of nanoshell nanolayers and nanoshell:donor:acceptor composites were also determined. The dopant QDs shifted the extinction maximum toward longer wavelengths. The maximum refractive indices values for layers with QDs were lower than those for pure P3HT:PC71BM. An increase in the roughness of the active layers with additives used in ternary solar cells was determined using surface morphology studies. The HOMO and LUMO levels of the studied nanodots were obtained by conducting UPS analysis, and then the energy graph of the solar cell was built. Impedance spectroscopy results showed increased conductivity of the active layer upon doping with quantum dots. Current–voltage characteristics and standard parameters of photovoltaic cells were simulated for two architectures, that is, with and without an additional layer. The architecture with the higher efficiency proved to be the one without the PEDOT:PSS layer. Results demonstrated that quantum dots are emerging materials for photovoltaics and are an essential component of research to develop organic solar cells.

## Supporting Information

File 1 Additional material from AFM studies.
