# Supplementary material for: CdSe/ZnS quantum dots as a booster in the active layer of distributed ternary organic photovoltaics
Source: Beilstein J Nanotechnol. 2024 Feb 2;15:144–56. doi: 10.3762/bjnano.15.14 (PMC10840543; doi:10.3762/bjnano.15.14)
Supplement: File 1 — Additional material from AFM studies. [file Beilstein_J_Nanotechnol-15-144-s001.pdf]

BEILSTEIN JOURNAL OF NANOTECHNOLOGY

Supporting Information

for

CdSe/ZnS quantum dots as a booster in the active layer of distributed ternary organic photovoltaics

Gabriela Lewińska, Piotr Jeleń, Zofia Kucia, Maciej Sitarz, Łukasz Walczak, Bartłomiej Szafraniak, Jerzy Sanetra and Konstanty W. Marszalek

Beilstein J. Nanotechnol. **2024,** *15,* 144–156. doi:10.3762/bjnano.15.14

Additional material from AFM studies

License and Terms: This is a supporting information file under the terms of the Creative Commons Attribution License (https://creativecommons.org/licenses/by/4.0). Please note that the reuse, redistribution and reproduction in particular requires that the author(s) and source are credited and that individual graphics may be subject to special legal provisions.
The license is subject to the *Beilstein Journal of Nanotechnology* terms and conditions: (https://www.beilstein-journals.org/bjnano/terms)

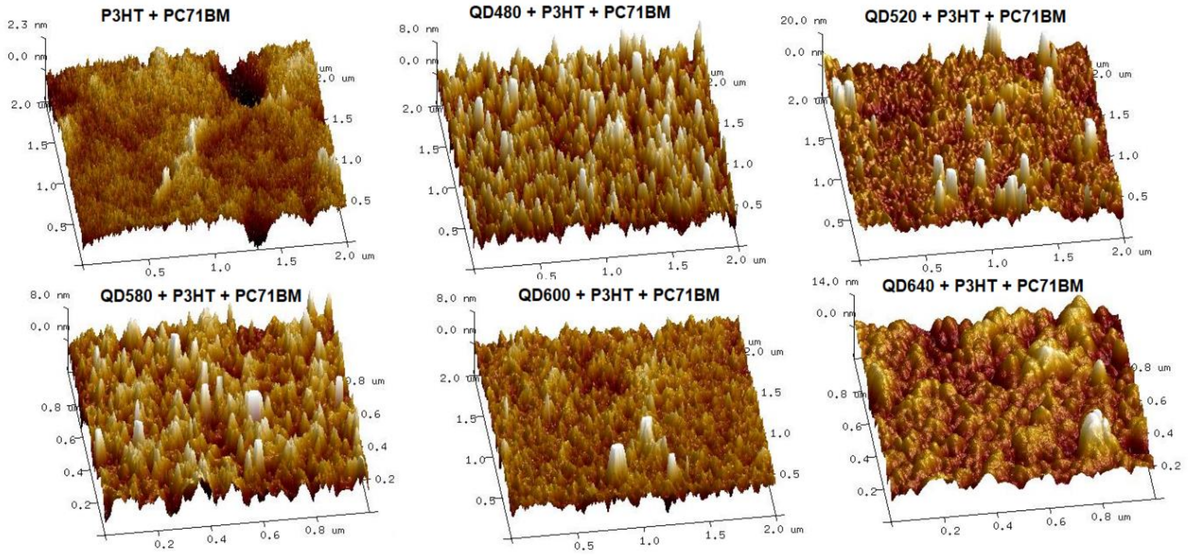

Figure S1: 3D AFM images of investigated quantum dots, P3HT and PCBM mixtures.
